# Female germline stem cells: aging and anti-aging

**DOI:** 10.1186/s13048-022-01011-2

**Published:** 2022-07-04

**Authors:** Wenli Hong, Baofeng Wang, Yasha Zhu, Jun’e Wu, Li Qiu, Shuyi Ling, Ziqiong Zhou, Yuqing Dai, Zhisheng Zhong, Yuehui Zheng

**Affiliations:** 1 Reproductive Health Department, Shenzhen Traditional Chinese Medicine Hospital, the Fourth Clinical Medical College of Guangzhou University of Traditional Chinese Medicine, Shenzhen, Guangdong 518000 People’s Republic of China; 2 Shenzhen University Health Science Center, Shenzhen, Guangdong 518000 People’s Republic of China; 3 ARTcenter, Shenzhen Hengsheng Hospital, Shenzhen, Guangdong 518000 People’s Republic of China

**Keywords:** Female germline stem cells, Stem cell aging, Ovarian aging, Aging related mechanisms, Anti-aging strategies

## Abstract

The delay of ovarian aging and the fertility preservation of cancer patients are the eternal themes in the field of reproductive medicine. Acting as the pacemaker of female physiological aging, ovary is also considered as the principle player of cancer, cardiovascular diseases, cerebrovascular diseases, neurodegenerative diseases and etc. However, its aging mechanism and preventive measures are still unclear. Some researchers attempt to activate endogenous ovarian female germline stem cells (FGSCs) to restore ovarian function, as the most promising approach. FGSCs are stem cells in the adult ovaries that can be infinitely self-renewing and have the potential of committed differention. This review aims to elucidate FGSCs aging mechanism from multiple perspectives such as niches, immune disorder, chronic inflammation and oxidative stress. Therefore, the rebuilding nichs of FGSCs, regulation of immune dysfunction, anti-inflammation and oxidative stress remission are expected to restore or replenish FGSCs, ultimately to delay ovarian aging.

## Introduction

Because populations around the world are aging fast, the issue of aging has become a growing concern. With aging, the body's immunity and regenerative response to damage decreases, leading to development of aging-related diseases. One of the underlying causes of senescence-induced functional decline is thought to be the depletion of in situ stem cell function. Changes in the overall environment, ecological niche, and stem cells themselves related to aging contribute to this decline. Therefore, restoring stem cell function at the cellular level may result in individual rejuvenation at the organismal level, which could shed light on the development of new solutions to age-related dysfunctions and diseases [1]. With the delay of the age of first childbearing and the population structure in the modern society, the ovarian aging and related problems are becoming more serious. Ovaries cease obviously earlier than the rest organs of the body [2], which in turn ushers the start aging of whole body. Along with the substantial extension of woman life span in modern society, the asynchronous aging of ovary and whole body becames a sharp conflict with pursueing better physical and psychological well-being in woman at advanced-age. At the contemporary era in which infertility is regareded as one of the three highest diseases in humans, infertility rates soar as high as 15–20% in developed countries. In China, infertility rate has increased from 3% to 15–18% in the past four dacades, of which about 40% is related to ovarian aging in female patients. In the whole population of childbearing age women, 1–3% of women reach pathological menopause, or pathological ovary function decline, also called premature ovarian failure (POF), before the age of 40 [3]. Due to longer life span and increasing infertility incidence, ovarian aging become a huge challenges to woman health of modern society. Therefore, delaying ovarian aging is a hot key node of preserving fertility of advanced age women, POF patients or cancer patients, improving women's health and quality of life, as well as coping with aging population structure!

### FGSCs aging and ovarian aging

The underlying mechanisms for the aging of the ovary are still poorly understood, partically because it is a complex biological process in which many factors interact internally and externally. Compared with the “evergreen” male testeis, female ovaries in advanced age women are more like “rotten root of old tree”. What makes this big different? The researchers believe that the FGSCs aging directly determines the ovarian aging. In phyicilogical conditions, when women reach their advanced age, the stem cells in their ovaries are exhausted, they face menopause and symptoms of hypoestrogene, while male enjoy their old age life without dramatic decline of their testes function. They still have the ability to father as long as their spouses are young enough.

### Evidence of FSCG's existence in ovaries

Adult stem cells are undifferentiated cells that exist in an adult individual and are present in various tissues and organs of the body. They can be infinitely self-renewing and have the potential of targeted differentiation in an organism, which not only play an important role in the organogenesis, but also have an amazing ability to maintain and sustain tissue organ regeneration [4]. The presence of testicular stem cells was first described over 40 years ago, and this is the source of the adult's continued sperm production and maintanance of "evengreen" testicular function. Whether mammal's ovaries have FGSCs to supplement the original follicle pool after birth has been debated for nearly one century. The earliest report to answer this debate dates back to the late nineteenth century by German autopsist Wilhelm Waldeyer, who was also famous for creating the concepts of “chromosome” and “neuron”. He hypothesized that adult female mammals lost the de novo oocyte renewal abilitybased on their work mainly of histological study that showed the absence of excess oocyte production on post-perinatal period. The hypothesis dominated the academic circles for long time [5]. In the early 1950s, Zuckerman declared that there was no proof that females formed new oocytes after birth, adhering the concept of a restricted (stationary) mammalian ovarian reserve had been the main dogma of the cycle [6]. Unstill recent years, more and more researchers reported that the ovarian surface epithelium contains germ cells that form follicles. The fate of such germ cells is full of uncertainty, since many of them are either lost during migration or undergo cell death [7, 8]. In 2004, Johnson discovered the consumption rate of non-atretic follicles was less than the atresia follicle forming rate in mice. Hence, they believe there are renew of follicles in mice and come up with FGSCs theory, which was a big challenge to ovarian follicle fixed theory [9]. Later, scientists isolate cells that could be subcultured in vitro and express the both stem and germ cell-specific protein markers in mice, adult mice, rats, and human ovarian tissue cortex respectively. Using a variety of methods, including stem cell culture and expansion, stem cell transplantation, genetic modification and gene editing, in vivo cell lineal tracking, the researchers confirmed existence of FGSCs in the postnatal ovaries in a variety of mammals, including humans, even old women ovarian surface epithelium, and observed that FGSCs had the ability to direct differentiation into eggs, continuously replenish follicle pools, and restore progeny to infertile model. FGSCs with GFP were transplanted into infertility mice, and both mature follicles with GFP and offspring with GFP were is covered obtained, which provided the most direct evidence of FGSCs existence [10–30]. Our team also successfully isolated FGSCs from the ovarian surface epithelium of mice. In our study, FGSCs could be stably passed on by generation, and could increase the infertile mice follicles number and successfully produce offspring after transplantation [31–33]. Having features of embryonic stem cells, OSE cell layer is considered as the "germ epithelium", in which it serves as the bipolar progenitor cell of oocytes and granulosa cells. In order to pinpoint the exact propotion of FGSCs in ovary, White's team evaluated that the population of FGSCs in mouse ovaries represented 0.014% ± 0.002% of the total cell population [17]. FGSCs expressed markers of both pluripotency stem cells and germ cells, such as Ddx4(VASA), Dppa3 (major maternal effect gene maintaining pluripotency), Prdm1 (early germ cells marker as transcriptional repressor), Pou5f1(POU Class 5 Homeobox 1), Dazl, Ifitm3, but not Nanog (pluripotency marker), Fig. la, Kit, Sycp3 and Zp3 [24]. Afer injecting adult FGSCs into fetal ovaries, Sharma found that foreign stem cell participatied various events during oogenesis and follicle assembly. In the intricate expriment, author confrimed the exitence of stem cell in adult ovaries and have the potential to form follice and oogenesis [34].

### Classification of FGSCs

FGSCs’ sizes vary considerably, ranging from 2–8 um [12, 18]. Several research groups reported the existence of two stem cell populations (VSELs and OSCs) in mice, rabbits, sheep, marmosets and human ovaries [19, 35, 36]. Several years later, Esmaeilian's group confirmed VSELs have the capacity to be differentiated into oocyte-like structures [37]. Evidence began to accumulate that two types of stem cells, resting stem cells and activated stem cells, exist in various organs of the adult body [38, 39]. Likewise, the two stem cell populations consist of relatively quiescent very small embryonic-like stem cells (VSELs) and their direct progeny, the "progenitor cells", called FGSCs. Being relatively quiescent, VSELs experience asymmetrical cell divisions to become progenitor cells that divide rapidly, expanding clonal offsprings through symmetrical cell divisions. These expanded progenitor cells form cysts, and ultimately differentiate into oocytes in the ovaries [35–37, 40–45]. Such stem/progenitor cells express follicle-stimulating hormone (FSH) receptors and are activated by FSH. VSELs maintain life-long homeostasis, and may survive radiotherapy and chemotherapy. Its impairing lead to host age-related senescence due to loss of function caused by impaired ecology, and the presence of overlapping pluripotency markers suggests that they may also be associated with epithelial ovarian cancer [46–50].

### FGSCs and remodeling of ovarian function

FGSCs were defined as committed progenitors, capable of renewing and differentiating into oocytes and remodeling of ovarian function. In 2009, Virant-Klun’s team isolated and obtained FGSCs in postmenopausal women, after induced differentiation in vitro, were detected to express zona pellucida 2 (ZP_2_),observed to have a polar—like structure, shaped like an oocyte [12]. In the same year, Zou and colleagues identified and isolated FGSCs in neonatal and adult mouse ovaries and cultured them in vitro. They found that they had the potential to proliferate and were passed on for over 60 generations, and successfully established mouse FGSCs lines. FGSCs, transfected with GFP, were transplanted into the ovaries of infertility model mice. Transplanted cells underwent oogenesis and the mice produced offspring that had the GFP transgene. These findings contribute to basic research into oogenesis and stem cell self-renewal and open up new possibilities for applications of FGSCs in biotechnology and medicine [13]. White’s team isolated and purified female germline stem cells from adult mouse ovaries using the FACS. They also isolated FGSCs from the ovaries of adult women, and transplanted them into the cortical layer of mouse ovaries after in vitro culture. Immature oocytes were found in the mouse ovaries, and ovarian granular cells were formed around the ovaries [17]. In 2012, Hu and his colleagues also separation FGSCs from mice, and added GSK3 inhibitor-Bio into the culture system to make FGSCs induced differentiation to oocytes. PCR and immunohistochemical results showed that stem cell markers Oct4 expression was decreased and oocyte markers were increased [16]. However, are active FGSCs available in postnatal mouse ovaries? By labeling small germ cells expressing Oct4, Guo's team tracked their fate for up to 4 months and observed for the first time the presence of FGSCs with active function in adult mouse ovaries [23]. In addition, Satirapod’s team reported that mouse FGSCs express E_2_ receptor-α (ERα). During oogenesis process, ERα has interaction with the E_2_ stimulated by retinoicacid gene 8 (Stra8) promoter, which can drive Stra8 expression [30]. These discoveries establish a critical physiological role for FGSCs in oogenesis. However, whether FGSCs can develop into functional oocytes in vitro and its characteristics have not been reported. Wu and her team used the 3D culture system to establish the ovarian organoid model derived from mouse FGSCs. Like a normal ovary, the ovaries are filled with follicles and secrete hormones. Through single-cell sequencing, Wu's team revealed that the ovarian organoids contained 7 cell groups, including germ cells, granulosa cells, follicular membrane cells and fibroblasts. Follicles isolated from ovarian organoids can develop into mature oocytes by in vitro culture. Mature oocytes can produce normal offspring by in vitro fertilization [51].

Accumalating evidence suggest FGSCs transplantation can prolongs fertility age, cure infertility, and recover ovarian function of cancer patients. Being evently matched function of primordial germ cells, ovarian pluripotential VSELs can be differentiated into an oocyte-like structure which may bypass many obstacles that ES/iPS cells therapy faced.

### Factors for FGSCs aging

#### Niche and FGSCs aging

Schofield presented stem cell niche hypothesis in 1978 [52]. The niche is the microenvironment for stem cells relay on and dwell. In gerenal, the niche is composed of extracellular matrix, niche cells, granulocytes, blood vessels, immune cells and secreted factors. According to the conventional view, stem cell aging leads to the senescence of organs. Its components are complicated and Its functional state can decide the subsistence of stem cells.

In the gonads of drosophila, the FGSCs niche could produce bone morphogenetic proteins (BMP), which act as ligands to FGSCs receptors and increase the expression level of BMP by inducing BMP signal cascade amplification [53]. Besides, the escort cells in the drosophila germinal stem cell niche directly affect FGSCs via GTPaseRho regulation and functional defect of Rho increase abnormal BMP level in the niche, leading to accumulation of undifferentiated single germ cells [54]. In consideration of that FGSCs niche structure and function in mammals are less clear than that of drosophila, we believe that the mammal niche is similar to the drosophila niche. The niche of ovaries in mammals maybe includes follicular membrane-stromal cells, granulosa cells, extracellular matrix, blood vessels, immune system-related cells and cytokines [55]. Bukovsky observed that niche of FGSCs formed during early embryonic development consist of nonspecific ovarian monocyte-derived cells(MDCs), T cells, and vascular endothelial cells, whereas nests of adult ovarian germinal stem cells consist of primary CD14 + MDCs, activated HLA-DR + MDCs and T cells [56]. Furthermore, after transplantation of ovarian tissue from senescent mice into the ovaries of young mice, the good status of donor primordial follicles with GFP were observed in the host mice; However, after transplanting ovarian tissue from young mice into older mice, ovarian tissue of the young mice were found to have reduced number of follicles and lack of mature follicles [57]. Recently, after transplantating stem cell from old mouse ovaries into young mouse ovary ovaries,Sharma found exitency of stem cell and differentiate, but fail to form follicle, which hint that we still know little about niche and the interaction between niche and stem cells [58]. Bhartiya and her team reported a series excellent expriments demonstrated that very small embryonic-like stem cells (VSELs), being dorment in most time, can survive oncotherapy, spontaneously differentiate into gametes after tranplantation, showing big potential to regenerate de novo functional gonads in the fufure [40, 44, 47]. The findings also imply that stem cell(VSELs)may altert niche’s habitability by some mechism so far we don’t know yet. Similiar to pluripotent stem cells, very small embryonic-like stem cells (VSELs) in adult gonads, developmentally equivalent to migratory primordial germ cells, can survives oncotherapy due to their quiescent nature [44, 59–61]. Unfortunately, the stem cell's residence– niche was impaired by oncotherapy [57, 62–64]. Transplanting niche cells (mainly refer to Sertoli or mesenchymal cells) can regenerate the non-functional gonads [65, 66]. This approach has esulted in the birth of fertile offspring in mice. Besides it's safty, this approach and strategy show a strong application prospect. It could be used as first line treatment for permanent restoring gonadal function in POF and cancer patients.

Accumulating evidence suggest FGSCs niche is the key link to ovarian failure. FGSCs niche might be more important than aging of FGSCs themselves.

#### Immune system,inflammatory factors and FGSCs aging

The immune system, composed of immune organs, immune cells and secreted factors, is essential for the body to defend itself against external damage. The immune system can be classified into innate and adaptive immunity. Adaptive immunity can be further categorized into cell-mediated immunity and humoral immunity. The immune system has three important functions.(1) immune defense function, which fights against the invasion of pathogenic microorganisms such as viruses, bacteria, andfungus; (2) immune homeostasis function, which removes aging and dead cells to maintain the body's juvenscence; (3) Immune surveillance functions, or in more understandable term, identifying, killing and eliminating mutated cells to prevent cancer. These functions are key to maintaining good health, while dysfunctional immune function contributes to disease. Beside the role in maintaining body homeostasis, the immune system also has a crucial role in regulating ovarian function. The immune system coordinates the development of the ovaries, follicle maturation, and ovulation. As early as 1970s, researchers devoted considerable effort to understanding the relationship between the ovary and the immune system. Sakakura and Nishizuka observed that the ovaries of thymus-free mice fail to mature after birth [67]. Russell et al. noted that the thymocytes of wild-type female mice can inhibit cyclophosphamide and X-ray induced superovulation [68, 69]. As early as 1979, Bukovsky and Presl proposed a hypothesis that the immune system is an important player in ovarian function [70]. Since then, this hypothesis has been supported by a growing number of studies. For example, thymosin injection in neonatal nude mice promotes maintenance of follicle-stimulating hormone and luteinizing hormone levels during later reproductive maturation, rescuing abnormal development of the ovary in nude mice [69]. Furthermore, it has been shown that the immune system can deteriorate with age, with a consequent decline in ovarian function [71]. These findings suggest a strong association between the immune system and the function of ovaries. Bukovsky put forward that the thymus was the largest immune organ in the body, and follicular depletion speed in mice with thymic removal was faster [70]. In ovaries, immune-related components, such as MDCs, T cell, and B cell, have significant effects on ovarian function maintenance. MDCs could stop cellular differentiation and keep cells in quiescence, in case of over-differentiation. However, due to immune system degeneration by age or diseases, ovaries occurred morphostasis and dysfunction, leading to premature ovarian failure or primary menopause [71]. Although the linkage between the functions of ovarian and the immune system was identified as early as 1970s, less focus has been placed on the relationship between the nests of FGSCs and the immune system. However, the fate of FGSCs mainly relies on immune cells and cytokines. There are many uncommitted MDCs and T cells in ovaries, which could recognize FGSCs. T cells differentiate into ovarian memory cells (OMC) and transmit ovarian information into lymph tissue. When niche degenerated, T cells would not produce OMC anymore. Therefore, symmetric and asymmetric division of FGSCs cannot be triggered [70–72].

Macrophages, play an importance role in maintaining the stable state of the stem cell niche. Macrophages produce numerous cytokines, both IL-10 and TNF-α, which speed up the clearance of aging erythrocytes and dead tissue. Therefore, macrophages may contribute to the senescence of the stem cell niche [73]. Furthermore, several cytokines, such as TNF-α and IL-1, can facilitate the processes of ovulation and angiogenesis. Animal model experiments have showed that TNF-α can facilitate vasculogenesis by increasing the expression of vascular endothelial growth factor in developing corpus luteum and inhibits angiogenesis in mature corpus luteum. Howere, this dual-regulatory mechanism of TNF-α remain to be elucidated [74]. Neverthless, the microenvironment of inflammatory can significantly impair the stem cell niche [75]. We found IL-2 and TNF-ɑ rose in senescent FGSCs while anti inflammatory factors TGF-βand IL-10 declined(unpulished). In the genetic level, the expression of MVH, Oct-4 and anti-aging gene SITR-1 and SIRT3 decreased, and aging gene P21 and P53 associated protein express increased. Those evidences indicated chronic inflammatory reaction in niche played an essential role in FGSCs senescence [55, 76–78]. Thus, related immune cells and molecules from the ovarian stem cell nests are engaged in the asymmetric/symmetric division of OGSCs, migration, primordial follicle and neo-granulosa cell formation, and follicle maturation.

#### Hypoxia, oxidative stress injury and FGSCs aging

**Fig.1:**
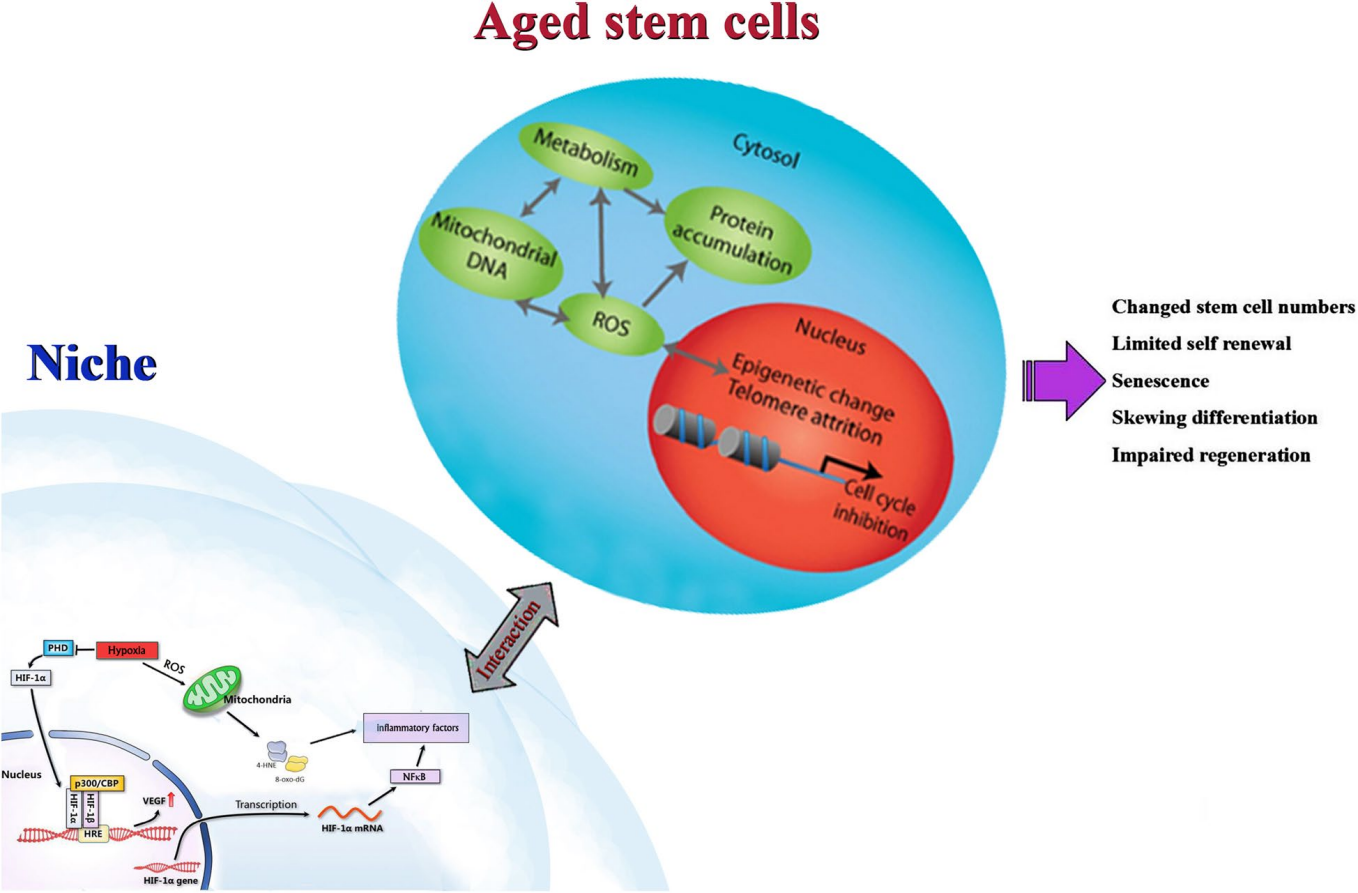
Hypoxia-induced chronic inflammation and oxidative stress on stem cell senescence. Hypoxia promotes the generation of inflammatory factors such as TNF-α, IFN7 and IL-6 through hypoxia-inducible factor-1 alpha (HIF-1 α), leading to chronic ovarian inflammation, and also induces excessive production of ROS, leading to the formation of DNA adducts such as 8-OXo-7, 8-dihydro-2'-deoxyguanine and 4-hydroxynonenal. Together with inflammatory factors, these two substances cause accumulation of damage to DNA, changes in epigenetics, abnormalities in gene expression, dysregulation of cellular signaling pathways, etc., ultimately leading to aging of cells and organs

As mentioned above, the stem cell niche determines stem cell aging and remained stem cell numbers. Stem cell niche, combined with exogenous microenvironment alterations, such as changes from oxgen tension, temperature, hormones or cytokines from blood supplement, results in estricted self-renewal, senesence, skewed differentiation and compromised regeneration.The endogenous mechanisms inducing aging include telomere shortening, DNA damage accumulation, abnormal gene expression, epigenetic alteration, abnormal cell signaling [1] (Fig. 1). In the exogenous microenvironment, special focus has to be placed on the role of hypoxia in inducing and accelerating stem cell aging. Hypoxia, the unbalance between oxygen supply and demand, is the primary culprit of oxidative stress and chronic inflammation. Unfortunately, ovary is a deeply hypoxic organ due to it’s unique structure and cell compositon. On the one hand, with the growth and progression of follicular oocytes and the proliferation and division of granulosa cells, the oxygen demand gradually increases. On the other hand, continuous ovulation results in the increase of fibrous connective tissue and the significant reduction of blood vessels in the ovary, which leads to the decrease of oxygen supply in the ovary, and the decrease of blood vessels and blood supply in the ovary with the increase of age. In addition, chronic, low-grade inflammatory response caused by repeated ovulation and the accompanying oxidative stress aggravate the imbalance between supply and demand, resulting in low oxygen concentration in the ovary to about 1.3%-5.5% [79, 80]. This may be the important reason that the speed of ovarian aging should be obviously faster than other organs by roughly 20–30 years earler. Molinari's transcriptomic analysis of human cumulus cells also revealed that hypoxia is a marker and an important determinant of follicular aging [81]. Hypoxia increases glycolysis metabolism,through hypoxia inducible factor-1 α (HIF-1 α), hypoxia activates monocytes/macrophages and TH1/TH17 cells through nuclear transcription factor NFκB (NFκB binding site exists in HIF-1 α promoter) and promotes the output of inflammatory factors like IL-6, IFN7 andTNF-α, resulting in chronic ovarian inflammation. Hypoxia also induced overproduction of ROS, which leading to damage to the ovaries from oxidative stress, oxidation of biomolecules including lipids, DNA and proteins,. Oxidative DNA damages, in turn, promote the formation of DNA adduct compounds such as 8-OXo-7, 8-dihydro-2 '-deoxyguanine and 4-hydroxynonenal. Both of them, together with inflammatory factors, cause the accumulation of DNA damage, abnormal gene expression, epigenetic alteration, dysregulation of cell signaling pathways and so on, leading to cell and organ aging. Many studies support a vicious cycle of oxidative stress-chronic inflammation: ROS increases due to oxidative stress, mediating through nod-like receptor 3 and NFκB to cause a range of inflammatory responses, for example, increased production of IL-1β, IL-6 and TNF-α, which in turn promote oxidative stress to accelerate aging [82]. Recently, Wang’ s team used high precision single-cell transcriptome sequencing technology for the first time to draw a compairing map of *cynomolgus* monkey ovarian cell aging and human ovarian cell It was found that aging leads to the imbalance of cell type-specific REDOX regulatory network in ovary and the decline of antioxidant capacity with aging, which is one of the main characteristics of ovarian aging in primates [3].

As mentioned above, inflammatory factors can cause FGSCs aging, and oxidative stress also has an essential role in the aging of FGSCs. The concept of oxidative stress (OS) came up by Sohal in 1990, defining an adverse stress reaction that redundancy reactive oxide species produced by an imbalance of oxidative and anti-oxidative systems exceed eliminating ability. ROS is a chemically reactive oxygen atom or group of atoms produced during cellular metabolism. It could bi-directionally regulate cellular proliferation and apoptosis, where proper ROS concentration contributed to the activation of proliferative growth factors and their transduced signal molecules [83]. On the other hand, OS, in overdose situations, induce stem cell injury and multiorgan failure. For example, too much ROS caused skeletal muscle stem cell necrosis as pathological muscle injury and decreased hematopoietic stem cell activation [84]. Meantime, OS induces accumulation of malondialdehyde and ROS decreased expression of the anti-oxidative enzyme, injury mitochondria, inhibition of ovarian granulosa cell development. It also could cause ovarian inflammation reaction and reduce ovarian function, leading to infertility [85]. Excessive oxidative stress damage has been reported in the ovaries of chemotherapy-induced senescent mice, and elimination of oxidative stress damage promotes survival [86, 87].

### The strategy for delaying FGSCs aging

#### Rebuilding the nichs of FGSCs

One of the major challenges facing researchers and clinicians is successfully implementation of ovarian tissue engineering techniques to reconstruct the ovarian microenvironment and promote the initiation and maturation of primitive follicles. Tilly is optimistic about the future that rebuilding the habitable microenvironment of FGSCs for elderly women so that they could develop into mature eggs. He believe that this techeque will show great value in the future of assisted fertility technology [29]. Since alginate beads were first used in humans as artificial pancreas carriers in the 1980s, alginate polymers, because of it’s non-toxic, good elasticity, moderate solubility, transparency, long-term duration after implantis widely used in medical transplantation treatments. The channels within the hydrogel can promote the host cell invasion and form new vascular system, similar to the extracellular matrix (ECM).The gel pore network of ECM allows the combined nutrients and wastes to be delivered to tissues in a natural way, showing broad application prospects in 3D cell culture in vitro and the development of artificial organs in vivo [88, 89]. Whether this technique can be used to reconstruct the microenvironment of FGSCs proliferation and differentiation is a topic of great significance and clinical application value. Recently, we selected alginate as the scaffold for FGSCs proliferation and differentiation, which can be connected with a variety of active substances such as melatonin, and promote the proliferation and differentiation of FGSCs by long-term and stable reconstruction of an ovarian microenvironment that is physiologically equivalent to that of healthy women of childbearing age, so as to rebuild the nichs of FGSCs and preserve female fertility (unpublished).

#### Anti-chronic inflammation and oxidative stress

Chronic inflammation and OS could trigger FGSCs aging, therefore, anti-chronic inflammation and OS drugs with fewer side effects and favorable biocompatibility are in consideration.

##### Resveratrol (RES)

RES, a natural component rich in grapes, mulberries, and other plants [90], can exert a variety of pharmacological functions, including anti-oxidation, anti-inflammatory, immune regulation, cell protection, anti-tumor, and anti-apoptosis effects [91–97]. RES effectively removes the accumulation of ROS. Rat follicular cells were shown to be free from cisplatin-induced oxidative toxicity after removal of excess ROS [98]. RES treatment can be used to improve the function of ovarian follicles by decreasing TNF-α level, confirmed by reducing the level of LH and the ratio of LH/FSH, which serve as indicators of ovarian function [99]. Jiang found that RES dramatically enhanced body weight and ovarian index, as well as follicle number and reduced follicular atresia in POF mice. Higher levels of Mvh, Oct4, SOD2, GPx and CAT were measured after in vivo and in vitro treatment with RES. RES therapy caused a significant decrease in the concentrations of TNF-α and IL-6 and an increase in IL-10 in the ovaries. In FGSCs, Mvh, Oct4 and SOD2 concentrations were higher and TNF-α, IL-6 and MDA concentrations were lower in the RES group. In summary, RES efficiently enhanced ovarian function and FGSCs in animal models of POF by reducing oxidative stress and inflammation, indicating that RES is a potential drug against POF by promoting the survival of FGSCs [87]. The therapeutic effect of RES on ovarian aging may depend on promoting the function of FGSCs and improving the ecological niche of FGSCs.

##### Chitosan oligosaccharide (COS)

 COS is an absorbable degradation of chitosan. It has been proved that COS had the functions of anti-oxidation, bacteriostasis, hypoglycemic and obesity contro l [100, 101]. Our studies demonstrated that when the mice were given chitosan oligosaccharide by gavage for one month, the visceral coefficients of ovaries, the number of ovarian follicles, estrogen level and immune function enhanced. Meantime, the expression of the FGSCs marker increased, which was dependent on the COS dose, and also found chitooligosaccharides could directly promote the proliferation of FGSCs in vitro (unpublished). Our findings indicate COS has protective effects on ovaries through directly promote the proliferation of OGSCs or indirectly by enhancing the immune system.

##### Proanthocyanidin (PC)

PC is indispensable flavonoids in the human nutrition diet derived from grape seeds and pericarps. It has antibiotic, antioxidative and anticancer effects [102]. By now, there were few reports about the direct influence of PC on FGSCs, but the improvement of PC on ovarian aging has been studied [103, 104].Therefore, it was speculated that the PC had a beneficial effect on FGSCs by its anti-oxidative stress and anti-aging, but further researches are needed.

#### The role of traditional chinese medicine

##### Traditional Chinese medicine

Most complications of ovarian aging are linked directly to ovarian hormonal deficiencies. Hormone replacement therapy (HRT) continues to perform a central role in the therapy of ovarian aging. However, HRT increases health risks for some patients, including breast cancer and cardiovascular disease [105]. As a result, numerous patients are switching to complementary and alternate medicine (CAM). As one major branch of CAM, traditional Chinese medicine (TCM), regarded as having less side effects, has been extensively used in China and in other countries to treat ovarian aging [106–108]. According TCM theory, ovarian aging diseases i are related to kidney, liver, spleen, heart, lung and other zang-organs’ mulfunction(we need to declare specifically that in the TCM theory, “kidney, liver, spleen, heart, lung”are not as same as the cencepts of morden medicine), espectially uterus mulfunction; dysregulation of thorough fare and conception vessels is the key link of ovarian aging pathogenesis.The etiology and pathogenesis concludes cold evil invasion, qi and blood deficiency, liver stagnation and qi stagnation, phlegm and blood stasis block, which eventually lead to meridian block, stagnation of qi and blood activities, zang-fu dysfunction. Therefore, the treatment of POF mainly starts from regulating throughfare and conception vessels, supplementing kidney and replenishing essence, smoothing liver and regulating qi, strengthening spleen and nourishing blood, promoting blood circulation and collaterals.

##### Acupuncture

Acupuncture is the treasure of Chinese culture and has been recognized, accepted, applied and popularized in the world [109]. At present, acupuncture has treated more than 800 kinds of diseases, and about 30% to 40% of them have significant curative effects [110]. In particular, acupuncture can significantly improve ovarian aging and ovarian function of POF and regulate menstrual cycle with clinical symptom improvement. Compared with hormone replacement therapy, acupuncture has good efficacy, lower recurrence rate, and less side effects, resultant earier accepted by patients and broader application prospects [1, 111–113]. Meridians and collaterals spread all over the body, linking interio organs and body surface, maintaining the body's interior stability. Chong ren, nexus of the twelve meridinans on the surface, crosslink the uterus in the deep part of body, therefore affecting menstrual activity. Acupuncture stimulations on throughfare and conception vessels, Du Ren meridinans can tonify kidney and essence, regulat liver and qi, invigorate spleen and nourishing blood, activat blood and dredge collaterals, stimulat the functions of viscera, achieving the dual purpose of regulation.

Although Chinese traditional medicine has been widely used in the treatment of ovarian aging due to its precise curative effect, the mechanism of its treatment of ovarian aging is still far from clear. Whether it is related to the regulation of the function of FGSCs deserves further study.

## Conclusion

Ovarian physiological or pathological failure results in the follicle reduction and terminating ovulation. The essence of the follicle reduction is the decreased number of FGSCs after follicles depletion, which is influenced by many factors like stem cell niche, inflammatory factors, hypoxia, oxidative stress injury. The immune cells and cytokine are key players of the stem cell niche. Immune dynfunction not only directly reduced ovarian resistance, due to FGSCs being more vulnerable to adverse external factors, but also indirectly hindered FGSCs proliferation by causing FGSCs niche defects. Therefore, exploring the mechanism of FGSCs aging is helpful in solving female infertility fundamentally in clinical practice. Heretofore, some studies have come up with solution that partially replenish exhausted FGSCs, via rebuilding the nichs of FGSCs, altering the microenvironment of chronic inflammation and oxidative stress. Whether the effect of Chinese traditional medicine on FGSCs in delaying ovarian aging remains to be further studied.

## Data Availability

Not applicable((review article).

## Abbreviations

FGSCs: Female germline stem cells
POF: Premature ovarian failure
VSELs: Very small embryonic-like stem cells
FSH: Follicle-stimulating hormone
ZP2: Zona pellucida 2
ERα: E2 receptor-α
Stra8: Stimulated by retinoicacid gene 8
BMP: Bone morphogenetic protein
MDCs: Monocyte-derived cells
OMC: Ovarian memory cell
HIF-1 α: Hypoxia inducible factor-1 α
OS: Oxidative stress
ECM: Extracellular matrix
RES: Resveratrol
COS: Chitosan oligosaccharide
PC: Proanthocyanidin
HRT: Hormone replacement therapy
CAM: Complementary and alternate medicine
TCM: Traditional Chinese medicine
